# Endemic Nephropathy Around the World

**DOI:** 10.1016/j.ekir.2016.11.003

**Published:** 2016-11-11

**Authors:** Fiona J. Gifford, Robert M. Gifford, Michael Eddleston, Neeraj Dhaun

**Affiliations:** 1 Pharmacology, Toxicology & Therapeutics, University/British Heart Foundation Centre for Cardiovascular Science, University of Edinburgh, Edinburgh, UK; 2 South Asian Clinical Toxicology Research Collaboration (SACTRC), University of Peradeniya, Peradeniya, Sri Lanka; 3 Department of Renal Medicine, Royal Infirmary of Edinburgh, Edinburgh, UK

**Keywords:** CKD*u*, chronic kidney disease, endemic nephropathy

## Abstract

There have been several global epidemics of chronic kidney disease of unknown etiology (CKD*u*). Some, such as Itai-Itai disease in Japan and Balkan endemic nephropathy, have been explained, whereas the etiology of others remains unclear. In countries such as Sri Lanka, El Salvador, Nicaragua, and India, CKD*u* is a major public health problem and causes significant morbidity and mortality. Despite their geographical separation, however, there are striking similarities between these endemic nephropathies. Young male agricultural workers who perform strenuous labor in extreme conditions are the worst affected. Patients remain asymptomatic until end-stage renal failure. Biomarkers of tubular injury are raised, and kidney biopsy shows chronic interstitial nephritis with associated tubular atrophy. In many of these places access to dialysis and transplantation is limited, leaving few treatment options. In this review we briefly describe the major historic endemic nephropathies. We then summarize the epidemiology, clinical features, histology and clinical course of CKD*u* in Mesoamerica, Sri Lanka, India, Egypt, and Tunisia. We draw comparisons between the proposed etiologies and supporting research. Recognition of the similarities may reinforce the international drive to establish causality and to effect prevention.

Chronic kidney disease (CKD) is common, and is a significant cause of morbidity and mortality globally.^1^ Low- and middle-income countries have seen an alarming rise in CKD over the past 20 years.^2^ Indeed, the prevalence in these countries has now overtaken that in many high-income countries. Furthermore, patients in these countries present with more severe CKD and at a younger age.^2^ Although these trends can largely be attributed to traditional risk factors such as diabetes and hypertension,^3^ a considerable proportion of CKD remains unexplained.^1^ This has been termed CKD of unknown etiology (CKD*u*). In general, CKD*u* is a diagnosis of exclusion, made when a patient fulfils the Kidney Disease Improving Global Outcomes (KDIGO) CKD criteria but without evidence of a recognized cause such as diabetes, hypertension, or glomerulonephritis.^4^ It should be noted that many population prevalence studies sample patients only at 1 time point, and therefore do not prove chronicity (as outlined in the KDIGO guidelines), which may lead to inaccurate prevalence rates.

There have been several global epidemics of unexplained kidney disease—Balkan endemic nephropathy (BEN), Itai-Itai disease in Japan, Mesoamerican nephropathy (MeN), and Sri Lankan CKD*u* (Table 1, Figure 1). Further epidemics are present in India, Egypt, and Tunisia, where robust research is currently lacking. Etiology has been established for Itai-Itai disease and BEN, with the help of international research collaboration. Unfortunately, despite ongoing collaboration, the etiology of CKD*u* elsewhere remains unknown.^5^ Furthermore, in many of these places, access to dialysis and transplantation is limited, magnifying the societal and economic burden of CKD and end-stage renal failure (ESRF).^1^ Recognizing the enormity of the problem, the World Health Organization (WHO) and the US Centers for Disease Control and Prevention (CDC) have taken an active interest in CKD*u*.^6^ In this review, we shall briefly summarize Itai-Itai disease and BEN, 2 forms of endemic nephropathy the etiologies of which were clarified in 1968 and 1993, respectively, following decades of research. Thereafter, we shall focus on endemic CKD that remains unexplained.

**Figure 1:**
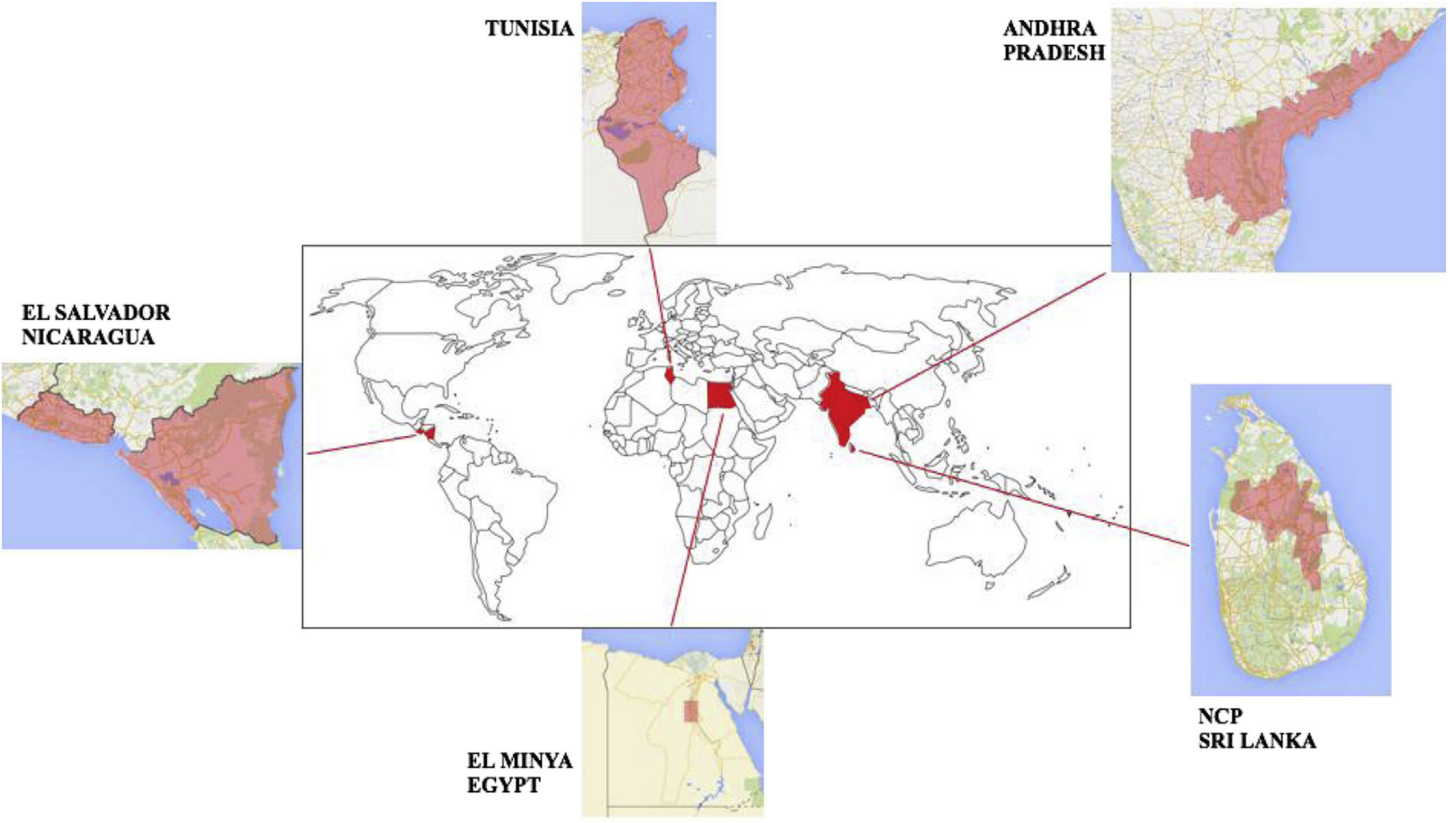
World map indicating areas with high prevalence of chronic kidney disease of unknown etiology (CKD*u*). Central map taken from Wikipedia (https://commons.wikimedia.org/wiki/File:The_World_map.png). Peripheral maps modified from Google maps (Tunisia, https://www.google.co.uk/maps/place/Tunisia; Andhra Pradesh, https://www.google.co.uk/maps/place/Andhra+Pradesh; Sri Lanka, https://www.google.co.uk/maps/place/Sri+Lanka; Egypt, https://www.google.co.uk/maps/place/Egypt; Nicaragua/El Salvador, https://www.google.co.uk/maps/place/Nicaragua). NCP, North Central Province of Sri Lanka.

**Table 1:** Comparison of current and previous chronic kidney disease of unknown etiology (CKD*u*)

	Itai-Itai	BEN	MeN	SL CKD*u*	Indian CKD*u*
Date first described	1912	1956	2002	Early 1990s	2010
Endemic areas	Jinzu river basin, Toyama, Japan	Danube region: Serbia, Bulgaria Croatia Romania Bosnia	Nicaragua El Salvador Costa Rica **Low-altitude** **Agricultural areas**	“Dry Zone” of Sri Lanka First noticed in NCP **Low altitude** **Agricultural areas** **Geographical foci of disease** **Low socio-economic status**	Uddanam coastal region, Andhra Pradesh **Agricultural areas** **Foci of disease Low socio-economic status**
Etiology confirmed?	Yes: 1968 Cd exposure	Yes: 1993 *Aristolochia sp.*	Unexplained	Unexplained	Unexplained
Characteristic clinical features	Postmenopausal women Bone pain, waddling gait	**Presents: fifth through sixth decade** M:F = 1:1 Tubular proteinuria Impaired concentrating capacity Tubular acidosis	**Presents: fourth through fifth decade** M:F = 5:1 **Asymptomatic until ESRF** **Recurrent “*Chistata”*: dysuria, frequency, sterile urine**	**Presents: fourth through fifth decade** M:F = 1:1.3 Severe disease more common in men **Asymptomatic until ESRF** **Recurrent dysuria, loin/back pain, sterile urine**	**Presents: fifth through sixth decade** M:F = 1:1 **Asymptomatic until ESRF**
Associated findings	Osteomalacia Proximal tubular dysfunction Tubular proteinuria	Urothelial carcinoma in 50%	**Normotensive at presentation** **Absent/mild proteinuria** **Elevated tubular biomarkers** Hyperuricemia Hypokalemia **Small kidneys on US**	**Normotensive at presentation** **Absent/mild proteinuria** **Elevated tubular biomarkers** Peripheral edema with late disease **Small kidneys on US**	**Normotensive at presentation** **Absent or mild proteinuria**
Renal histology	**Interstitial fibrosis** **Tubular atrophy** **Glomerular ischemia**	**Interstitial fibrosis** **Tubular atrophy** Aristolactam (AL)-DNA adducts in renal cortex	**Interstitial fibrosis** **Tubular atrophy** **Glomerulosclerosis** (despite normal BP) **Chronic glomerular ischaemia** Little vasculopathy	**Interstitial fibrosis** **Tubular atrophy** **Glomerulosclerosis** (55% hypertensive at biopsy) **Glomerular collapse** Moderate vasculopathy	**Interstitial fibrosis** **Tubular atrophy** Normal glomeruli
Frequently reported risk factors	Water source: Jinzu river basin Consumed contaminated crops	Consumption of wheat contaminated by Aristolochia sp.	**Occupation: sugarcane** **Heat stress** **Agrochemical exposure** **Heavy metal exposure** **Genetic predisposition** **Alcohol “Lija” consumption**	**Agricultural workers** Resident in dry zone ≥5 yrs **Heat stress** **Agrochemical exposure** **Heavy metal exposure** **Genetic predisposition** **Alcohol/betel/tobacco**	**Agricultural workers** **Heat stress** **Agrochemical exposure** **Heavy metals** **Genetic predisposition**

Highlighted in bold are the features common across different endemic nephropathies. BEN, Balkan endemic nephropathy; BMI, body mass index; BP, blood pressure; Cd, cadmium; CKD*u*, chronic kidney disease of unknown etiology; ESRF, end-stage renal failure; F, females; GFR, glomerular filtration rate; M, males; MeN, Mesoamerican nephropathy; NCP, North Central Province (Sri Lanka); SL, Sri Lankan; US, ultrasound.

### Itai-Itai Disease

From 1910 to the 1960s, wastewater from a mine near the Jinzu river basin in Toyama, Japan, polluted water and rice paddies with heavy metals, including cadmium (Cd). As early as 1912, patients reported bone pain, muscle weakness, and renal failure. In 1968, the Japanese Ministry of Health and Welfare identified this as “*Itai-Itai”* (“ouch-ouch”) disease from chronic Cd exposure. Cd has an elimination half-life of 10 to 30 years and accumulates in the kidney.^7^ Bone pain (hence the name), waddling gait, osteomalacia, and irreversible proximal tubular dysfunction led to a severe, disabling condition.^8^ Evidence revealed a dose−effect relationship between blood Cd level (an effective estimate of whole body Cd burden) and ESRF.^9^ Histology from the few reported kidney biopsies revealed interstitial fibrosis, tubular atrophy, and ischemic glomerular lesions.^10^ High concentrations of Cd were found in soil, rice, and in pathology specimens of individuals with *Itai-Itai*.^11^ A large 16-year follow-up study identified a dose-related increase in overall age-adjusted mortality, and mortality related to cardiovascular and kidney disease.^12^

Cd-induced nephropathy still exists today. Exposure is primarily through contaminated food, smoking, or occupational contact. The WHO set a “safe exposure level” in 1981, based on the relationship between urinary Cd excretion and renal dysfunction in occupationally exposed workers.^13^ Renal dysfunction was thought to be unlikely at urinary Cd concentrations of ≤10 μg Cd/g creatinine. Later work revealed this to be a gross underestimate of risk.^7^ Cd-induced renal disease was found in 10% of an environmentally exposed Belgian population at urinary concentrations of only 2 to 3 μg Cd/g creatinine.^14^ A study of 902 Swedish battery workers identified urinary β_2_ microglobulin (a measure of tubular dysfunction) as an effective screening tool for early identification of Cd nephrotoxicity.^15^ Prompt recognition and subsequent avoidance can prevent progression to ESRF.^15^, ^16^ No chelating agent has been identified, so renal replacement therapy remains the mainstay of treatment.

### Balkan Endemic Nephropathy

BEN was first recognized in the 1950s in rural villages along the Danube River. Those affected presented in their sixth decade with tubular proteinuria, impaired concentrating capacity, and reduced glomerular filtration rate (GFR).^17^ Additional features included tubular acidosis, glycosuria, and aseptic leucocyturia. Hypertension was a late feature and edema rare. Kidney biopsy revealed interstitial fibrosis with tubular atrophy, and up to 50% of patients had a concomitant urothelial carcinoma.^18^ Progression to ESRF was slow. Affected villages were situated next to unaffected ones, and familial clustering suggested possible genetic susceptibility.^19^ Males and females were equally affected, but children did not develop the disease.

In 1969, Lijec Vjesn first proposed that ingestion of flour contaminated with seeds from *Aristolochia clematitis* might be the cause of BEN.^20^ Approximately 25 years later, Vanherwegham *et al.* published a case series of 9 Belgian women who developed “Chinese herb nephropathy” after ingesting slimming remedies containing aristolochic acid.^21^ Similar renal histology and concurrent urothelial malignancy strongly suggested that *Aristolochia* plants, found growing among wheat in the endemic area, were responsible for BEN. More recently, this was confirmed when aristolactam (AL)−DNA adducts were demonstrated in the renal cortex of individulals with BEN and their urothelial tumors.^22^ Specific adenine:thymine to thymine:adenine transversion of the *p53* tumor suppressor gene was identified.^22^ Similar mutations and AL-DNA adducts have been identified in Taiwan, which has the world’s highest prevalence of urothelial malignancy and where use of aristolochic acid containing herbal remedies is widespread.^23^ Cases have also been reported in Australia, North America, and Europe. *Aristolochia* species continue to be used in herbal remedies worldwide. There is no specific treatment, so therapy is largely supportive, aiming to delay disease progression.

### Mesoamerican Nephropathy

#### Epidemiology

Mesoamerican nephropathy (MeN) has emerged as a leading cause of morbidity and mortality in low-altitude coastal areas of Nicaragua and El Salvador, with additional foci in Costa Rica and Guatemala.^24^ WHO data for 2012 showed a CKD mortality rate of 54 deaths per 100,000 population in Nicaragua and 36 per 100,000 in El Salvador, compared to 10 per 100,000 in the United States.^25^ CKD mortality increased ∼3-fold in Nicaragua between 1990 and 2009 and ∼7-fold in El Salvador.^26^ A community survey in El Salvador found that 18% of adults had CKD, of whom more than half had no traditional risk factors.^27^ Prevalence varies conspicuously with occupation; those affected are predominantly young male agricultural workers.^27^ Sugarcane seed cutters have the highest prevalence, although other “hot occupations” such as port workers, miners, and cotton and construction workers are also affected.^28^ Increased urinary biomarkers of tubular dysfunction (neutrophil gelatinase-associated lipocalin (NGAL) and *N*-acetyl-β-d-glucosaminidase [NAG]) in Nicaraguan adolescents from high-risk areas suggest that kidney injury may start in childhood. However, population reference values are unknown, so these results should be interpreted with caution.^29^

#### Clinical Features

Individuals affected complain of dysuria, frequency, urgency, and chills, collectively termed “*chistata*.” They have leucocyturia, although urine culture results are rarely positive.^30^ These episodes are often misdiagnosed as urinary tract infections and treated with (potentially nephrotoxic) aminoglycosides.^31^ Serum creatinine rises indolently, and persons affected usually present at ESRF. Histopathology is outlined in Table 1.

In a cross-sectional study of 284 Nicaraguan workers, estimated GFR (eGFR) and urinary biomarkers of kidney injury were measured prior to and during *zafra*, the 5-month period of sugarcane harvest.^30^ The authors compared different roles in the industry—cane cutter, seed cutter, irrigator, driver, seeder, agrochemical applicator, and factory worker. Cane and seed cutters had significantly lower late-*zafra* eGFR compared to individuals of other occupations, and their mean fall in eGFR during *zafra* was 5 to 7 ml/min/1.73 m^2^ greater. Urinary NGAL increased significantly during *zafra* among cane cutters. Moreover, late-*zafra* NGAL and NAG levels were negatively associated with eGFR. Workers who reported *chistata* had significantly lower eGFR and higher NGAL concentrations. Proteinuria remained low in all affected individuals. A recent longitudinal study by Wesseling *et al.* supports these findings.^32^

#### Etiological Hypotheses

##### Heat Stress

The etiology of MeN is likely multifactorial (Figure 2). The 2nd International Workshop on the Epidemic of MeN in 2015 emphasized the growing evidence for a causal role of strenuous work in intense heat with inadequate rehydration.^5^ A recent review has also articulated the role that global warming might play in the upsurge of CKD*u* in affected regions.^33^ Intense heat and strenuous work are common to those most at risk for MeN. However, CKD*u* is not observed in similar agricultural communities of developing countries in other tropical regions. Moreover, heat-associated acute kidney injury (AKI) is uncommon in developed countries but, when present, tends to accompany multi-organ injury.

**Figure 2:**
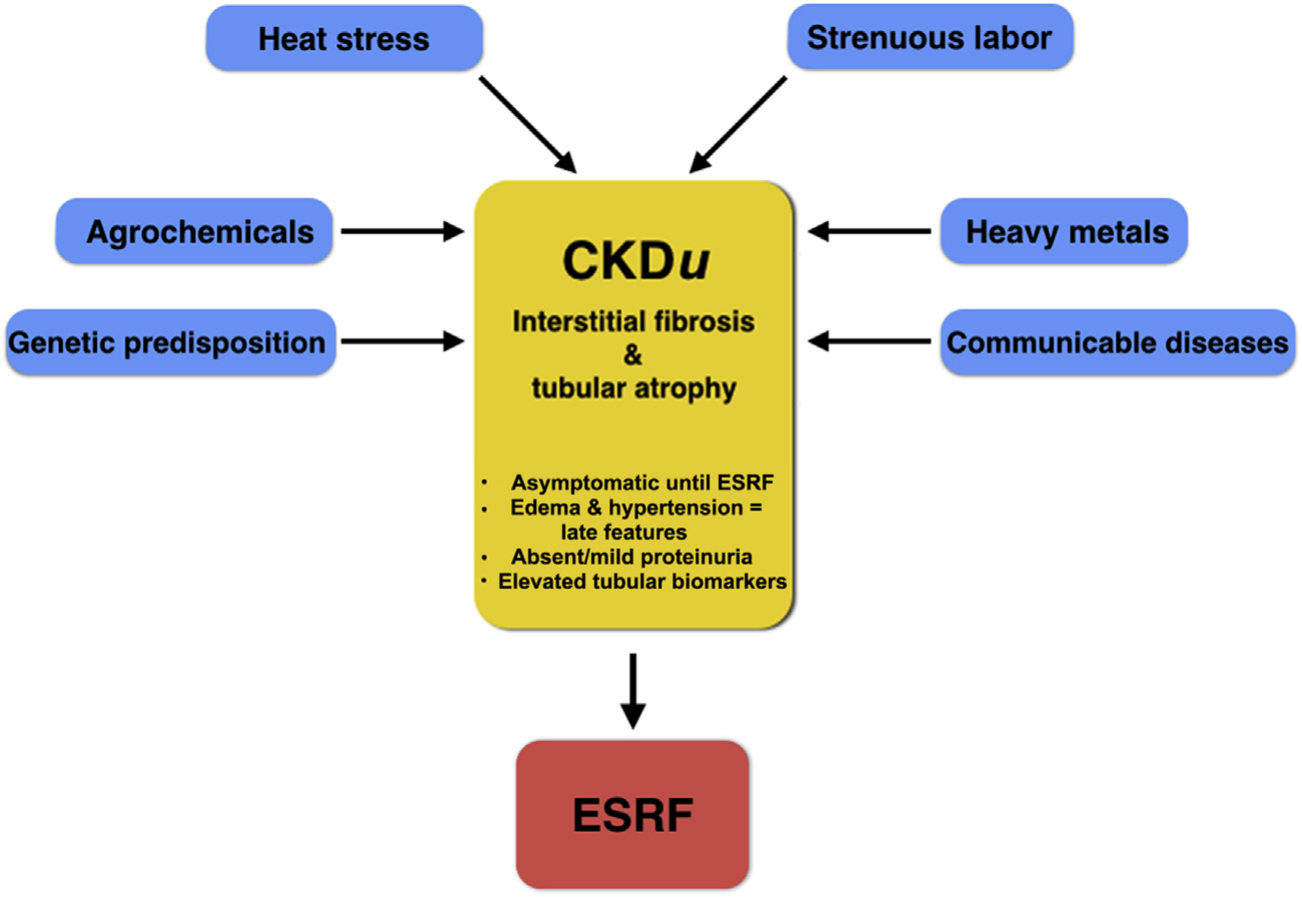
Multifactorial etiology of chronic kidney disease of unknown etiology (CKD*u*). ESRF, end-stage renal failure.

Roncal-Jimenez *et al.* postulated that dehydration-induced increases in urinary osmolality activate the aldose reductase pathway, converting glucose to fructose. In proximal tubules, fructose is metabolized by fructokinase to urate, oxidants, and inflammatory mediators, causing tubular injury.^34^ Workers chew sugarcane and rehydrate with fructose-rich drinks, exacerbating the problem. In support of this theory, recurrent heat-induced dehydration led to cortical urate accumulation, reduced GFR, proximal tubular injury, and fibrosis in mice.^34^ Strenuous exercise in hot climates causes lactic acid production and subclinical rhabdomyolysis, exacerbating hyperuricemia.^35^ Under such acidic conditions, urinary urate can exceed its solubility and form microcrystals.^36^ Indeed, urate crystalluria was identified in sugarcane workers, which may explain *chistata*.^36^ It should be noted, however, that although hyperuricemia is common in MeN, marked uricosuria is not universally demonstrated.^37^

##### Agrochemicals

Agrochemicals are used extensively throughout Nicaragua and El Salvador, and workers are often highly exposed.^38^ These may damage kidneys made vulnerable by heat-stress during *zafra*. Animal studies have identified dose-dependent and exposure duration-dependent kidney damage with specific pesticides including 2-4-dichlorophenoxyacetic acid (2,4-D), carbofuran, and dicrotophos.^39^, ^40^, ^41^ Data on the nephrotoxic effects of pesticides in humans are limited. A review of Nicaraguan pesticide use revealed no association between the 36 pesticides tested and CKD.^42^ However, there was a strong association between AKI and exposure to 2,4-D or glyphosate, the 2 most widely used herbicides in Nicaragua.^42^ Glyphosate was the most frequently used herbicide in the United States 10 years ago and, until recently, was used widely in Sri Lanka and El Salvador.^43^ It is recognized to cause kidney injury.^44^ A large US prospective clinical study showed no association between glyphosate exposure and ESRF^45^; however, an association between cumulative, general agrochemical exposure and increased ESRF was identified.^45^, ^46^ This relationship was especially marked in those who reported multiple doctor visits or hospitalizations due to agrochemical poisoning, suggesting that recurrent high-level exposure may lead to irreversible kidney damage.^45^

##### Heavy Metals

Heavy metals such as Cd, uranium, arsenic, and lead are known nephrotoxins.^16^, ^47^, ^48^ They contaminate water and soil in MeN-affected regions, although only at concentrations considered to be nontoxic.^16^, ^30^ Large volumes of contaminated water, consumed to replace exceptional fluid losses during *zafra*, may lead to a high total filtered load and potentially result in heavy metal nephrotoxicity. Conversely, the association between water consumption and renal insufficiency may simply highlight that individuals experiencing repeated episodes of dehydration then consume more water, or it may reflect a urinary concentrating defect secondary to tubular injury.^24^ *Lija*, a locally produced, unregulated rum, is another potential source of heavy metal and agrochemical exposure. Reports suggest that *Lija* is prepared in industrial containers previously containing pesticides. One small study identified a dose-dependent relationship between *Lija* consumption and reduced eGFR.^24^

##### Communicable Diseases

Leptospirosis is common and often subclinical in agricultural workers.^30^ Leptospirosis-induced AKI is nonoliguric with tubular dysfunction followed by reduced GFR.^49^ Renal histology reveals an acute interstitial nephritis with tubular necrosis.^50^ Theoretically, recurrent infection could contribute to CKD*u.* but it is unlikely to be independently responsible. Recurrent leptospirosis has been shown to cause CKD in other mammals, although not yet in humans.^51^

### Sri Lankan CKD*u*

#### Epidemiology

Contemporaneous to the MeN epidemic, a dramatic rise in CKD*u*, occurred in the North Central Province of Sri Lanka (NCP). First recognized in the early 1990s, recent estimates are that almost 20,000 persons have died of CKD*u*.^52^ A WHO community-based screening study revealed a prevalence of 13% in males and 17% in females.^6^ More severe disease was more common in men who had been resident in low-altitude farming communities in the dry zone for 5 years (Table 1).^6^ This may explain why previous studies, based on hospital attendance, identified a greater prevalence of CKD*u* in males,^53^ in keeping with MeN. Moreover, the CKD*u* problem is not confined to NCP. It is now the seventh leading cause of death nationally.^54^ Strikingly, many published studies aim to prove or disprove a single etiological factor, rather than address the complex interplay of insults likely to underlie etiology. The recent introduction of a CKD*u* patient registry should aid epidemiological research in the future.^55^

#### Clinical Features

Patients typically present in their fifth decade with ESRF. The mean age of diagnosis has fallen since the introduction of community screening.^56^ Early CKD*u* is largely asymptomatic, although patients describe recurrent dysuria with back pain and sterile urine. Anemia, hypertension, and edema are late features.

In 2012 Jayatilake *et al.* proposed a unifying definition for Sri Lankan CKD*u*: namely, an albumin-to-creatinine ratio ≥30 mg/g, a normal glycosylated hemoglobin (HbA_1c_ <6.5%) not on treatment for diabetes, blood pressure <160/90 mm Hg (or <140/90 mm Hg on antihypertensive medication), and no history of kidney disease or snake bite.^6^ Defined diagnostic criteria are essential for meaningful research. Unfortunately, this definition is likely to be underrepresentative, as proteinuria is mild or absent in early disease.^57^

Urinary tubular markers such as α_1_-microglobulin and NGAL are elevated in early CKD*u*, and steadily rise with disease progression.^57^, ^58^ Similarly, urinary kidney injury molecule−1 (KIM-1) may represent an early marker of disease.^59^ These tests represent more sensitive screening tools, although their cost prevents widespread use. A potential alternative may be calculation of the ratio of urinary albumin to total protein, which has been shown to be 99% specific for biopsy-proven primary tubulointerstitial disease.^60^ These tests should be considered in future study designs. The pathological findings of Sri Lankan CKD*u* are outlined in Table 1.

#### Etiological Hypotheses

##### Genetic Predisposition

There are discrete regions of high prevalence in a mosaic pattern that could represent a genetic predisposition. Certainly, family history has repeatedly been shown to have a strong association with CKD*u*.^61^ Recent whole-exome sequencing revealed a possible rare variant in the *KCNA10* gene, which encodes for a voltage-gated potassium channel found in proximal tubular cells, that could predispose to disease.^62^

##### Contaminated Water

Drinking from shallow wells increases CKD*u* risk.^63^, ^64^ Conversely, local residents who consume spring water have a low prevalence.^63^ Well water levels fluctuate with those of nearby canals, suggesting that the ground water table is recharged from irrigation and reservoir systems with significant potential for contamination. Mice fed with extracts of cyanobacteria (bluegreen algae) from endemic area reservoirs developed acute tubular necrosis, but not interstitial nephritis.^65^ Field work >6 hours per day, sun exposure, consumption of <3 L water per day, and history of malaria have been identified as Sri Lankan CKD*u* risk factors. Drinking pretreated water had significant protective effects.^66^

##### Agrochemicals

In the 1960s, the “green revolution” saw the introduction of high-yield seeds, chemical fertilizers, and pesticides. Further progress in the 1990s saw the introduction of the mini-tractor and agricultural mechanization.^67^ Agrochemicals are overused in Sri Lanka, and poor safety compliance leads to marked exposure.^68^, ^69^ The relationship between pesticide exposure and CKD*u* risk has been shown repeatedly.^6^, ^70^ Jayasumana *et al*. implicated glyphosate, suggesting that glyphosate−metal complexes could be responsible.^71^ Theoretically, the hard water in endemic areas could convert glyphosate to solid complexes of magnesium, calcium, and arsenic that are highly insoluble and poorly absorbed.^72^ However, the researchers showed urinary glyphosate and heavy metal excretion to be higher in both individuals with CKD*u* and healthy controls in endemic areas, compared to controls in nonendemic areas.^73^ Interestingly, CKD*u* is not observed in the northern province of Sri Lanka, despite harsher environmental conditions than NCP. It has been suggested that this may be linked to a ban on agrochemicals in this area during the conflict (1980–2009) because of the potential for use in improvised explosive devices.^67^

##### Heavy Metals

In 2008, Bandara *et al.* reported high Cd concentrations in reservoirs serving CKD*u* households (as well as in soil, rhizomes, rice, and milk).^74^ They were unable to reproduce these findings in 2010,^75^ and others later contested their results.^6^, ^76^ Significant seasonal variation in toxin concentration is likely. Unfortunately, the authors did not publish the dates of sample collection.

Nanayakkara *et al.* showed urinary Cd excretion to be lower in CKD*u* patients (and their unaffected relatives) compared to controls.^58^ This was consistent with the findings of Bandara *et al.* (2008), who suggested that an inability to express the urinary chelating protein metallothionine led to both reduced urinary Cd concentration and increased tubular damage.^58^ More recent collaboration between the Sri Lankan Ministry of Health and WHO has again implicated Cd. However, they found *increased* urinary Cd in CKD*u* patients compared to healthy controls from both endemic and nonendemic areas, demonstrating a dose−effect relationship between urinary Cd and CKD stage.^6^ The absence of controls with CKD of known etiology makes the applicability of this finding uncertain. Conflicting results can be explained, in part, by heterogeneity of study design, control selection, and diverse means of assaying Cd. Inclusion criteria and CKD*u* definition (where defined) also vary.

One study has suggested that Sri Lankan agrochemicals and fertilizers can be contaminated by arsenic.*^77^* A recent systematic review supported an association between arsenic exposure and proteinuria, but reported mixed evidence for any association with CKD.^78^ Arsenic contamination of well water was reported to be high; however, this finding has not been reproduced, and urinary arsenic levels do not vary across regions.^6^ Jayasumana *et al.* suggested that a high calcium concentration in endemic area ground water may exacerbate arsenic toxicity.^71^

Chandrajith *et al.* suggested that hard water could enhance the cytotoxic properties of fluoride.^79^ Undeniably, fluoride levels in drinking water from endemic regions are above WHO safe levels for tropical countries^80^, ^81^; however, adjoining farms have not seen significant CKD*u*.^82^ Other theories include the formation of fluoro-aluminium complexes when boiling fluoride-rich water in aluminum kettles (often constructed from discarded car engines). Normal serum aluminum concentrations in CKD*u* patients suggest little association.^6^

##### Communicable Disease

Ochratoxin A, a mycotoxin known to cause interstitial fibrosis, has been identified in many foods in NCP, but at levels below European safety limits. Higher urinary Ochratoxin A levels found in CKD*u* patients and their unaffected relatives compared with Japanese controls are of uncertain significance.^83^ Traditional (ayuverdic) medications have also been implicated. Acute interstitial nephritis has been reported after ingestion of the herbal medicine Dioscorea quinqueloba.^84^ However, use of traditional medication is not limited to endemic areas.^85^ Interestingly, *Aristolochia* spp were found in 66 ayuverdic prescriptions investigated by the WHO.^55^ Moreover, as described in MeN, leptospirosis is endemic in Sri Lanka and has been linked to CKD*u*.^86^ Hantavirus is another important zoonotic disease that is spread through the inhalation of aerosolized rodent excrement. It presents with clinical features similar to those of leptospirosis (flu-like illness and fever) and is known to cause AKI. Although it has been implicated in the etiology of CKD*u*, hematuria is almost always present in hantavirus-induced AKI, and progression to CKD has not been proved.^86^

### Indian CKD*u*

In 2010, the first Indian CKD registry report was published with data from 52,273 adults.^87^ Although the most common cause of CKD was diabetes (31%), a significant proportion (16%) had CKD*u*. Geographical disease foci were recognized, with prevalence reaching ∼40% in coastal regions of Andhra Pradesh.^51^ Affected individuals are young and of low socio-economic status. Men and women are affected equally. In keeping with Sri Lankan CKD*u* and MeN, patients remain asymptomatic until late in the disease, have absent or mild hypertension, and have little or no proteinuria. Farming communities are severely affected, and local residents believe that manual labor performed in severe heat is responsible, alongside liberal pesticide use.^88^ When biopsied, histology reveals interstitial fibrosis, tubular atrophy, and a variable lymphocytic peritubulitis.^89^ Unfortunately, creatinine estimation is not standardized across India, CKD diagnostic criteria vary, and the biopsy rate is unknown, making large-scale research challenging.^90^

Interestingly, an association between *CYP1A1* polymorphisms and Indian CKD*u* suggests a possible genetic predisposition.^91^ Further work has linked polymorphisms of xenobiotic metabolizing enzymes with increased pesticide accumulation and reduced eGFR.^92^ Although some studies suggest that water contamination by Cd-containing manures and lead-containing pesticides may be responsible,^93^ this is not a universal finding.^94^

### Egyptian CKD*u*

CKD is also emerging as a serious health problem in Egypt. Although national statistics are not available, ESRF prevalence increased from 250 to 367 per million population in Egypt’s El Minya Governorate between 2002 and 2007.^95^ A cross-sectional study of dialysis patients revealed that 13% had diabetic nephropathy, 21% hypertension, and 27% CKD*u.*^96^ Drinking from tube wells, family history of renal disease, inhabiting a rural area, and pesticide exposure were all associated with increased CKD*u* risk. The authors suggest that CKD*u* develops when genetically predisposed individuals are exposed to an environmental trigger.^97^

### Tunisian CKD*u*

In Tunisia, a chronic interstitial nephritis of unknown etiology with striking similarities to CKD*u* was first described in 2003.^98^ After an insidious course, patients present in their fourth or fifth decade with ESRF.^99^ Food contamination with ochratoxin A is widespread,^100^ and serum ochratoxin levels are higher in CKD*u* patients than in controls.^101^ Despite this, not all who are heavily exposed develop CKD, suggesting a genetic predisposition.^100^

### Recommendations and Conclusions

CKD*u* is a serious global health problem. The past 5 years have seen increased awareness and worldwide collaboration, which are pivotal in the attempt to control the epidemic. The current body of evidence supports the theory of heat stress, arduous exercise, and inadequate hydration, in a genetically predisposed population or those exposed to a further insult such as agrochemicals. If this is accurate, global warming will inevitably lead to even greater disease burden in these, and other, vulnerable populations. There remains a need for concise diagnostic criteria, not only in MeN and Sri Lankan CKD*u*, but also in other endemic nephropathies. Similarly, validation of and funding for more sensitive biomarkers of disease would allow early detection and an opportunity to try to slow disease progression. Wider use of renal biopsy would provide useful diagnostic information. Further evaluation of the cardiovascular impact of CKD*u* would enable more effective primary prevention. Fundamentally, many of the proposed etiological factors are potentially preventable with appropriate education, health and safety regulations, and public health intervention. Improved working conditions and the provision of adequate, safe drinking water are essential. A recent intervention in El Salvador revealed that the provision of accessible water, mobile shaded rest areas, and scheduled rest periods not only reduced heat stress symptoms, but increased worker productivity.^102^ Moreover, the WHO and the Food and Agriculture Organization of the United Nations (FAO) have made strong recommendations including quality control for imported fertilizers, compulsory provision of personal protective equipment for agrochemical sale and use, tighter regulation on sales of agrochemicals thought to be nephrotoxic, improved health education, and financial assistance for both individuals with CKD*u* and researchers. Despite significant resistance, the sale of glyphosate was recently banned in both Sri Lanka and El Salvador.^103^

## Disclosure

All the authors declared no competing interests.

## References

[1] Jha V., Garcia-Garcia G., Iseki K. (2013). Chronic kidney disease: global dimension and perspectives. Lancet.

[2] Mills K.T., Xu Y., Zhang W. (2015). A systematic analysis of worldwide population-based data on the global burden of chronic kidney disease in 2010. Kidney Int.

[3] Global Burden of Disease Study 2013 Collaborators (2015). Global, regional, and national incidence, prevalence, and years lived with disability for 301 acute and chronic diseases and injuries in 188 countries, 1990–2013: a systematic analysis for the Global Burden of Disease Study 2013. Lancet.

[4] Kidney Disease: Improving Global Outcomes (KDIGO) CKD Work Group (2013). KDIGO 2012 Clinical Practice Guideline for the Evaluation and Management of Chronic Kidney Disease. Kidney Int Supp.

[5] Mesoamerican Nephropathy: Report From the Second International Research Workshop on MeN. Available at: http://www.regionalnephropathy.org/wp-content/uploads/2016/08/MeN-2015-Scientific-Report-high-resolution_final.pdf. Accessed December 20, 2016.

[6] Jayatilake N., Mendis S., Maheepala P. (2013). Chronic kidney disease of uncertain aetiology: prevalence and causative factors in a developing country. BMC Nephrol.

[7] Jarup L., Berglund M., Elinder C.G. (1998). Health effects of cadmium exposure—a review of the literature and a risk estimate. Scand J Work Environ Health.

[8] Emmerson B.T. (1970). Ouch-Ouch Disease: The Osteomalacia of Cadmium Nephropathy. Ann Intern Med.

[9] Jarup L., Persson B., Elinder C.G. (1995). Decreased glomerular filtration rate in solderers exposed to cadmium. Occup Environ Med.

[10] Bonnell J.A., Ross J.H., King E. (1960). Renal lesions in experimental cadmium poisoning. Br J Ind Med.

[11] Kobayashi E., Okubo Y., Suwazono Y. (2002). Association between total cadmium intake calculated from the cadmium concentration in household rice and mortality among inhabitants of the cadmium-polluted Jinzu River basin of Japan. Toxicol Lett.

[12] Nishijo M., Morikawa Y., Nakagawa H. (2006). Causes of death and renal tubular dysfunction in residents exposed to cadmium in the environment. Occup Environ Med.

[13] Bernard A., Buchet J.P., Roels H. (1979). Renal excretion of proteins and enzymes in workers exposed to cadmium. Eur J Clin Invest.

[14] Buchet J.P., Lauwerys R., Roels H. (1990). Renal effects of cadmium body burden of the general population. Lancet.

[15] Jarup L., Elinder C.G. (1994). Dose-response relations between urinary cadmium and tubular proteinuria in cadmium-exposed workers. Am J Ind Med.

[16] Hotz P., Buchet J.P., Bernard A. (1999). Renal effects of low-level environmental cadmium exposure: 5-year follow-up of a subcohort from the Cadmibel study. Lancet.

[17] Stefanovic V., Djukanovic L., Cukuranovic R. (2011). Beta2-microglobulin and alpha1-microglobulin as markers of Balkan endemic nephropathy, a worldwide disease. Ren Fail.

[18] Ferluga D., Hvala A., Vizjak A. (1991). Renal function, protein excretion, and pathology of Balkan endemic nephropathy. III. Light and electron microscopic studies. Kidney Int Suppl.

[19] Toncheva D., Dimitrov T. (1996). Genetic predisposition to Balkan endemic nephropathy. Nephron.

[20] Ivic M. (1969). [Etiology of endemic nephropathy]. Lijec Vjesn.

[21] Vanherweghem J.L., Depierreux M., Tielemans C. (1993). Rapidly progressive interstitial renal fibrosis in young women: association with slimming regimen including Chinese herbs. Lancet.

[22] Grollman A.P., Jelakovic B. (2007). Role of environmental toxins in endemic (Balkan) nephropathy. October 2006, Zagreb, Croatia. J Am Soc Nephrol.

[23] Lai M.N., Wang S.M., Chen P.C. (2010). Population-based case-control study of Chinese herbal products containing aristolochic acid and urinary tract cancer risk. J Natl Cancer Inst.

[24] Sanoff S.L., Callejas L., Alonso C.D. (2010). Positive association of renal insufficiency with agriculture employment and unregulated alcohol consumption in Nicaragua. Ren Fail.

[25] WHO. Age-standardized death rate, by cause—kidney diseases estimates for 2000-2012. Available at: http://apps.who.int/gho/data/view.main. Accessed December 10, 2015.

[26] World Health Organization. Disease and injury country estimates. Availabe at: http://www.who.int/healthinfo/global_burden_disease/estimates_country/en/. Accessed December 20, 2016.

[27] Orantes C.M., Herrera R., Almaguer M. (2011). Chronic kidney disease and associated risk factors in the Bajo Lempa region of El Salvador: Nefrolempa study, 2009. MEDICC Rev.

[28] Ramirez-Rubio O., McClean M.D., Amador J.J. (2013). An epidemic of chronic kidney disease in Central America: an overview. J Epidemiol Community Health.

[29] Ramírez-Rubio O., Amador J.J., Kaufman J.S. (2016). Urine biomarkers of kidney injury among adolescents in Nicaragua, a region affected by an epidemic of chronic kidney disease of unknown aetiology. Nephrol Dial Transplant.

[30] McClean M., Amador J.J., Laws R. (2012). Biological sampling report: investigating biomarkers of kidney injury and chronic kidney disease among workers in Western Nicaragua.

[31] Brooks D., McClean M. (2012). Boston University investigation of chronic kidney disease in Western Nicaragua, 2009-2012.

[32] Wesseling C., Aragón A., González M. (2016). Kidney function in sugarcane cutters in Nicaragua—a longitudinal study of workers at risk of Mesoamerican nephropathy. Environ Res.

[33] Glaser J., Lemery J., Rajagopalan B. (2016). Climate change and the emergent epidemic of CKD from heat stress in rural communities: the case for heat stress nephropathy. Clin J Am Soc Nephrol.

[34] Roncal Jimenez C.A., Ishimoto T., Lanaspa M.A. (2014). Fructokinase activity mediates dehydration-induced renal injury. Kidney Int.

[35] Knochel J.P., Dotin L.N., Hamburger R.J. (1974). Heat stress, exercise, and muscle injury: effects on urate metabolism and renal function. Ann Intern Med.

[36] Roncal-Jimenez C., Garcia-Trabanino R., Barregard L. (2016). Heat stress nephropathy from exercise-induced uric acid Crystalluria: a perspective on Mesoamerican nephropathy. Am J Kidney Dis.

[37] Garcia-Trabanino R., Jarquin E., Wesseling C. (2015). Heat stress, dehydration, and kidney function in sugarcane cutters in El Salvador—a cross-shift study of workers at risk of Mesoamerican nephropathy. Environ Res.

[38] Rodriguez T., Younglove L., Lu C. (2006). Biological monitoring of pesticide exposures among applicators and their children in Nicaragua. Int J Occup Environ Health.

[39] Poovala V.S., Huang H., Salahudeen A.K. (1999). Role of reactive oxygen metabolites in organophosphate-bidrin-induced renal tubular cytotoxicity. J Am Soc Nephrol.

[40] Kaur B., Khera A., Sandhir R. (2012). Attenuation of cellular antioxidant defense mechanisms in kidney of rats intoxicated with carbofuran. J Biochem Mol Toxicol.

[41] Uyanikgil Y., Ates U., Baka M. (2009). Immunohistochemical and histopathological evaluation of 2,4-dichlorophenoxyacetic acid-induced changes in rat kidney cortex. Bull Environ Contam Toxicol.

[42] McClean M., Laws R., Rubio O.R. (2010). Industrial hygiene/occupational health assessment: evaluating potential hazards associated with chemicals and work practices at the Ingenio San Antonio (Chichigalpa, Nicaragua).

[43] United States Environmental Protection Agency. United States Environmental Protection Agency Pesticides Sales Market Estimates, 2007. Available at: https://www.epa.gov/sites/production/files/2015-10/documents/market_estimates2007.pdf. Accessed November 29, 2016.

[44] Mohamed F., Endre Z.H., Pickering J.W. (2016). Mechanism-specific injury biomarkers predict nephrotoxicity early following glyphosate surfactant herbicide (GPSH) poisoning. Toxicol Lett.

[45] Lebov J.F., Engel L.S., Richardson D. (2016). Pesticide use and risk of end-stage renal disease among licensed pesticide applicators in the Agricultural Health Study. Occup Environ Med.

[46] Hsu C.Y., Iribarren C., McCulloch C.E. (2009). Risk factors for end-stage renal disease: 25-year follow-up. Arch Intern Med.

[47] Goyer R.A. (1989). Mechanisms of lead and cadmium nephrotoxicity. Toxicol Lett.

[48] Hsueh Y.M., Chung C.J., Shiue H.S. (2009). Urinary arsenic species and CKD in a Taiwanese population: a case-control study. Am J Kidney Dis.

[49] Daher E.D.F., de Abreu K.L.S., da Silva Junior G.B. (2010). Leptospirosis-associated acute kidney injury. J Bras Nefrol.

[50] Arean V.M. (1962). The pathologic anatomy and pathogenesis of fatal human leptospirosis (Weil’s disease). Am J Pathol.

[51] Wesseling C, Crowe J, Hogstedt C, et al. Report from the First International & Research Workshop on MeN. Costa Rica: Program on Work, Environment and Health in Central America (SALTRA) and Central American Institute for Studies on Toxic Substances (IRET). Universidad Nacional (UNA): Universidad Nacional Costa Rica; 2013.

[52] Annual Health Bulletin—Sri Lanka Ministry of Health Medical Statistics Unit; 2012. Available at: http://www.health.gov.lk/enWeb/publication/AHB-2012/Annual%20Health%20Bulletin%20-%202012.pdf. Accessed December 20, 2016.

[53] Nanayakkara S., Komiya T., Ratnatunga N. (2012). Tubulointerstitial damage as the major pathological lesion in endemic chronic kidney disease among farmers in North Central Province of Sri Lanka. Environ Health Prev Med.

[54] Alwis K. (2013). Chronic kidney disease—when scientists disagree.

[55] Mendis S. (2012). Progress report: chronic kidney disease of uncertain etiology (CKDu), Sri Lanka.

[56] Noble A., Amerasinghe P., Manthrithilake H. (2014). Review of literature on chronic kidney disease of unknown etiology (CKDu) in Sri Lanka.

[57] Redmon J.H., Elledge M.F., Womack D.S. (2014). Additional perspectives on chronic kidney disease of unknown aetiology (CKDu) in Sri Lanka—lessons learned from the WHO CKDu population prevalence study. BMC Nephrol.

[58] Nanayakkara S., Senevirathna S.T., Karunaratne U. (2012). Evidence of tubular damage in the very early stage of chronic kidney disease of uncertain etiology in the North Central Province of Sri Lanka: a cross-sectional study. Environ Health Prev Med.

[59] De Silva P.M., Mohammed Abdul K.S., Eakanayake E.M. (2016). Urinary biomarkers KIM-1 and NGAL for detection of chronic kidney disease of uncertain etiology (CKDu) among agricultural communities in Sri Lanka. PLoS Negl Trop Dis.

[60] Smith E.R., Cai M.M., McMahon L.P. (2012). The value of simultaneous measurements of urinary albumin and total protein in proteinuric patients. Nephrol Dial Transplant.

[61] Nanayakkara S., Senevirathna S.T., Abeysekera T. (2014). An integrative study of the genetic, social and environmental determinants of chronic kidney disease characterized by tubulointerstitial damages in the North Central Region of Sri Lanka. J Occup Health.

[62] Nanayakkara S., Senevirathna S.T., Parahitiyawa N.B. (2015). Whole-exome sequencing reveals genetic variants associated with chronic kidney disease characterized by tubulointerstitial damages in North Central Region, Sri Lanka. Environ Health Prev Med.

[63] Jayasekara J.M., Dissanayake D.M., Adhikari S.B. (2013). Geographical distribution of chronic kidney disease of unknown origin in North Central Region of Sri Lanka. Ceylon Med J.

[64] Jayasumana C., Paranagama P., Agampodi S. (2015). Drinking well water and occupational exposure to herbicides is associated with chronic kidney disease, in Padavi-Sripura, Sri Lanka. Environ Health.

[65] Dissananyake DM, Jayasekera JMKB, Ratnayake P, et al. The short term effect of cyanobacterial toxin extracts in mice kidney. Peradeniya University Research Sessions. Available at: http://www.pdn.ac.lk/purse/Proceedings/2011/helth2/h2_8.pdf. Accessed November 29, 2016.

[66] Siriwardhana E.A.R.I.E., Perera P.A.J., Sivakanesan R. (2015). Dehydration and malaria augment the risk of developing chronic kidney disease in Sri Lanka. Indian J Nephrol.

[67] Jayasumana C, Orantes C, Herrera R, et al. Chronic interstitial nephritis in agricultural communities: a worldwide epidemic with social, occupational and environmental determinants [e-pub ahead of print]. *Nephrol Dial Transplant.* pii:gfw346. 10.1093/ndt/gfw346 28186530

[68] Gupta A. (2012). Pesticide use in South and South-East Asia: environmental public health and legal concerns. Am J Environ Sci.

[69] Gifford R., Siribaddana S., Forbes S. (2015). Endocrine-disrupting chemicals and the diabetes epidemic in countries in the WHO South-East Asia region. Lancet Diabetes Endocrinol.

[70] Peiris-John R.J., Wanigasuriya J.K., Wickremasinghe A.R. (2006). Exposure to acetylcholinesterase-inhibiting pesticides and chronic renal failure. Ceylon Med J.

[71] Jayasumana C., Gunatilake S., Senanayake P. (2014). Glyphosate, hard water and nephrotoxic metals: are they the culprits behind the epidemic of chronic kidney disease of unknown etiology in Sri Lanka?. Int J Environ Res Public Health.

[72] Dharmawardana M.W.C., Amarasiri S.L., Dharmawardene N. (2014). Chronic kidney disease of unknown aetiology and ground-water ionicity; study based on Sri Lanka. Environ Geochem Health.

[73] Jayasumana C., Gunatilake S., Siribaddana S. (2015). Simultaneous exposure to multiple heavy metals and glyphosate may contribute to Sri Lankan agricultural nephropathy. BMC Nephrol.

[74] Bandara J.M., Senevirathna D.M., Dasanayake D.M. (2008). Chronic renal failure among farm families in cascade irrigation systems in Sri Lanka associated with elevated dietary cadmium levels in rice and freshwater fish (Tilapia). Environ Geochem Health.

[75] Bandara J.M., Wijewardena H.V., Bandara Y.M. (2011). Pollution of River Mahaweli and farmlands under irrigation by cadmium from agricultural inputs leading to a chronic renal failure epidemic among farmers in NCP, Sri Lanka. Environ Geochem Health.

[76] Rango T., Jeuland M., Manthrithilake H. (2015). Nephrotoxic contaminants in drinking water and urine, and chronic kidney disease in rural Sri Lanka. Sci Total Environ.

[77] Jayasumana C., Fonseka S., Fernando A. (2015). Phosphate fertilizer is a main source of arsenic in areas affected with chronic kidney disease of unknown etiology in Sri Lanka. Springerplus.

[78] Zheng L., Kuo C.-C., Fadrowski J. (2014). Arsenic and chronic kidney disease: a systematic review. Curr Environ Health Rep.

[79] Chandrajith R., Dissanayake C.B., Ariyarathna T. (2011). Dose-dependent Na and Ca in fluoride-rich drinking water—another major cause of chronic renal failure in tropical arid regions. Sci Total Environ.

[80] Dharmaratne R.W. (2015). Fluoride in drinking water and diet: the causative factor of chronic kidney diseases in the North Central Province of Sri Lanka. Environ Health Prev Med.

[81] Wasana H.M.S., Aluthpatabendi D., Kularatne W.M.T.D. (2016). Drinking water quality and chronic kidney disease of unknown etiology (CKDu): synergic effects of fluoride, cadmium and hardness of water. Environ Geochem Health.

[82] Jayasumana M.A.C.S., Paranagama P.A., Amarasinghe M.D. (2012). Is hard water an etiological factor for CKDu?. First International Research Workshop on MeN.

[83] Desalegn B., Nanayakkara S., Harada K.H. (2011). Mycotoxin detection in urine samples from patients with chronic kidney disease of uncertain etiology in Sri Lanka. Bull Environ Contam Toxicol.

[84] Kim H.Y., Kim S.S., Bae S.H. (2014). Acute interstitial nephritis induced by Dioscorea quinqueloba. BMC Nephrol.

[85] Wanigasuriya K.P., Peiris-John R.J., Wickremasinghe R. (2007). Chronic renal failure in North Central Province of Sri Lanka: an environmentally induced disease. Trans R Soc Trop Med Hyg.

[86] Gamage C.D., Sarathkumara Y.D. (2016). Chronic kidney disease of uncertain etiology in Sri Lanka: are leptospirosis and hantaviral infection likely causes?. Med Hypotheses.

[87] Rajapurkar M.M., John G.T., Kirpalani A.L. (2012). What do we know about chronic kidney disease in India: first report of the Indian CKD registry. BMC Nephrol.

[88] Chavkin S. (2012). Mystery in the Fields: Verdant Terrain Conceals Clues to the Cause of a Fatal Kidney Disease. https://cloudfront-files-1.publicintegrity.org/documents/pdfs/MysteryintheFields.pdf.

[89] Machiraju R., Yaradi K., Gowrishankar S. (2009). Epidemiology of udhanam endemic nephropathy. J Am Soc Nephrol.

[90] Varma P.P. (2015). Prevalence of chronic kidney disease in India—where are we heading?. Indian J Nephrol.

[91] Siddarth M., Datta S.K., Ahmed R.S. (2013). Association of CYP1A1 gene polymorphism with chronic kidney disease: a case control study. Environ Toxicol Pharmacol.

[92] Siddarth M., Datta S.K., Mustafa M. (2014). Increased level of organochlorine pesticides in chronic kidney disease patients of unknown etiology: role of GSTM1/GSTT1 polymorphism. Chemosphere.

[93] Bawaskar H.S., Bawaskar P.H., Bawaskar P.H. (2010). Chronic renal failure associated with heavy metal contamination of drinking water: a clinical report from a small village in Maharashtra. Clin Toxicol (Phila).

[94] Reddy D.V., Gunasekar A. (2013). Chronic kidney disease in two coastal districts of Andhra Pradesh, India: role of drinking water. Environ Geochem Health.

[95] El Minshawy O. (2002). End stage renal disease in El Minia Governorate (Central Egypt): an epidemiological Study. J Egypt Soc Nephrol.

[96] El Minshawy O. (2011). End stage renal disease in El-Minia Governorate, Egypt: data of the year 2007. Nephro-Urol Mon.

[97] Kamel E.G., El Minshawy O. (2010). Environmental factors incriminated in the development of end stage renal disease in El-Minia Governorate, Upper Egypt. Int J Nephrol Urol.

[98] Abid S., Hassen W., Achour A. (2003). Ochratoxin A and human chronic nephropathy in Tunisia: is the situation endemic?. Hum Exp Toxicol.

[99] Hmaissia Khlifa K., Ghali R., Mazigh C. (2012). Ochratoxin A levels in human serum and foods from nephropathy patients in Tunisia: where are you now?. Exp Toxicol Pathol.

[100] Creppy E.E., Moukha S., Bacha H. (2005). How much should we involve genetic and environmental factors in the risk assessment of mycotoxins in humans?. Int J Environ Res Public Health.

[101] O'Brien E., Dietrich D.R. (2005). Ochratoxin A: the continuing enigma. Crit Rev Toxicol.

[102] Bodin T., Garcia-Trabanino R., Weiss I. (2016). Intervention to reduce heat stress and improve efficiency among sugarcane workers in El Salvador: phase 1. Occup Environ Med.

[103] Sustainable Pulse. Sri Lanka's new president puts immediate ban on glyphosate herbicides. Available at: http://sustainablepulse.com/2015/05/25/sri-lankas-new-president-puts-immediate-ban-on-glyphosate-herbicides/#.WD4FkvFshaU. Accessed May 25, 2015.

